# Combined Genetic Biomarkers Confer Susceptibility to Risk of Urothelial Bladder Carcinoma in a Saudi Population

**DOI:** 10.1155/2017/1474560

**Published:** 2017-02-27

**Authors:** Nasser Attia Elhawary, Anmar Nassir, Hesham Saada, Anas Dannoun, Omar Qoqandi, Ammar Alsharif, Mohammed Taher Tayeb

**Affiliations:** ^1^Department of Medical Genetics, Faculty of Medicine, Umm Al-Qura University, P.O. Box 57543, Mecca 21955, Saudi Arabia; ^2^Department of Molecular Genetics, Medical Genetics Center, Faculty of Medicine, Ain Shams University, Cairo 11566, Egypt; ^3^Department of Surgery, Faculty of Medicine, Umm Al-Qura University, Mecca 21955, Saudi Arabia; ^4^Department of Urology, King Abdullah Medical City Specialist Hospital, Mecca 21955, Saudi Arabia; ^5^Division of Internal Medicine, Al-Noor Specialist Hospital, Mecca 21955, Saudi Arabia

## Abstract

We evaluated the associations between seven single nucleotide polymorphisms and susceptibility to urothelial bladder carcinoma (UBC) in a Saudi population. Genomic DNA was taken from buccal cells of 52 patients with UBC and 104 controls for genotyping of GSTT1, GSTM1, rs4646903, rs1048943, TP53 rs1042522, rs1801133, and rs1801394 using PCR and TaqMan® assays. The rs1801133 and rs1801394 variants showed strong associations with UBC (OR = 2.3, *P* = 0.0002; OR = 2.6, *P* = 0.0001, resp.). Homozygosity of Pro72 conferred a significant double risk in cases compared with controls (30.8% versus 15.4%), but the homozygote Arg/Arg had no effect on risk. Genotypic combinations of GSTM1/GSTT1, rs4646903/rs1048943, and rs1801133/rs1801394 exhibited significant linkage with the disease (*χ*^2^ = 10.3, *P* = 0.006; *χ*^2^ = 13.9, *P* = 0.003; and *χ*^2^ = 20.4, *P* = 0.0004, resp.). The GSTM1 and rs1042522Arg and rs1801394G variant alleles were more frequent in current smokers with UBC (52.4%, 52.5%, and 64.3%, resp.) than were the corresponding wild-types. Despite some variants having only a slight effect on UBC risk, the interaction effect of combined genetic biomarkers—or even the presence of one copy of a variant allele—is potentially much greater. Perhaps more studies regarding next-generation genetic sequencing and its utility can add to the risk of UBC.

## 1. Background

The International Human Genome Sequencing Project [1] and the International HapMap Project [2] have generated considerable data on genetic variants and candidate genes for many diseases, including cancer. Urinary bladder cancer (BC) is the second most frequently diagnosed genitourinary cancer worldwide. In Saudi Arabia, the five-year prevalence is 11.6 per 100,000 men and 2.8 per 100,000 women. There have been 14.1 million new cancer cases, 8.2 million cancer deaths, and 32.6 million individuals living with cancer worldwide [3]. Almost 83% of all diagnosed BCs are cases of urothelial bladder carcinoma (UBC) (http://www.scr.org); one-third of UBC is classified as invasive UBC with a very high risk of distant metastases [4]. Aggressive metastatic UBC is untreatable and has a five-year survival rate around 6%. After endoscopic resection, 10–15% of noninvasive UBC will be upstaged into invasive UBC progressing to recurrence, and the patients acquire additional genetic mutations [5].

A growing number of genes regulating enzymes involved in xenobiotic and folate metabolism have been investigated, giving rise to increased knowledge of allelic variants. The impact of polymorphic variants of glutathione* S*-transferases (GSTs), cytochrome P450s (CYPs), methylenetetrahydrofolate reductase (MTHFR), and methionine synthase reductase (MTRR) on BC risk might be reflected by an association with the frequency of somatic mutations in the TP53 gene [6].

Several polymorphisms of CYP1A1 have been described and current information can be found on the Human CYP allele nomenclature website (http://www.cypalleles.ki.se). Two functional nonsynonymous polymorphisms have been reported to be associated with risk of BC [7, 8]. The CYP1A1 gene regulates the P450-1A1 enzyme that converts polycyclic aromatic hydrocarbons, such as the procarcinogen benzo[*a*]pyrene, into benzo[*a*]pyrene diol epoxide carcinogens. These carcinogens, which might be associated with BC in aluminum workers exposed to polycyclic aromatic hydrocarbons and in coal gasification workers exposed to aromatic amines, can be converted in vivo into more hydrophilic active derivatives by the CYP450 enzyme superfamily [9]. The rs1048943 allele results in an alteration of the functional protein and thus increases enzyme activity [10].

Both GSTM1 (MIM 138350) and GSTT1 (MIM 600436) exhibit genetic polymorphisms in populations displaying a large percentage of homozygous gene deletions “null-variant.” As GSTs are involved in several chemical carcinogens, such as benzo[*a*]pyrene diol epoxide [11, 12], and cellular detoxification, it has been reported that all GSTs enzymes might evolve to protect cells against reactive oxygen metabolites. Inherited loss of both alleles of GSTM1 or GSTT1 is a risk factor for tumors such as bladder, breast, oral, lung, and head and neck cancers (http://www.cancerindex.org/geneweb) [13, 14].

Polymorphisms in genes encoding the folate metabolizing enzymes MTHFR and MTRR might be linked to the etiology of cancer disorders. Deficient folates can lead to decreased DNA methylation and perhaps induce genomic instability or repression of the protooncogenes MTHFR and MTRR [15–17], but few articles have focused on the association between MTHFR or MTRR genes and risk of BC [16, 18, 19]. MTHFR (MIM 607093; 1p36.22) catalyzes the irreversible conversion of 5,10-methylenetetrahydrofolate into 5-methyltetrahydrofolate, which serves as a substrate for the remethylation of homocysteine to methionine with the subsequent synthesis of* S*-adenosylmethionine (SAM). Cobalamin (vitamin B12) is a cofactor in this reaction, and methionine synthase reductase (MIM 602568; 5p15.31) is required for the maintenance of MTR in its active state. Methionine is then converted into SAM, and DNA methyltransferases transfer the methyl group from SAM to the DNA. The common polymorphism MTRR 66A>G (rs1801394) results in lower enzyme activity [20] and is associated with homocysteine elevation. This mechanism may promote DNA hypomethylation, which is a common feature in early carcinogenesis [21].

The tumor suppressor protein* p53* (TP53, MIM 191170) is a sequence-specific transcription factor that works to maintain the integrity of the genome. On its induction in response to DNA damage, TP53 promotes cell cycle arrest in G1/S regulation point and initiates apoptosis if DNA damage proves to be irreparable [22]. An allelic nonsynonymous polymorphism of the TP53 gene (Pro72Arg) can play an important role in the carcinogenesis of many tumors, including urinary BC. This missense change (rs1042522) not only causes TP53 to no longer function as a tumor suppressor but also can affect the development of tumors [23]. It has been found that 72Arg has up to a 15-fold higher apoptotic ability than Pro72 in both inducible cell lines and cells with endogenous TP53 that is homozygous for each of the variants [24].

Historically, the Saudi population is highly heterogeneous and consists of indigenous Asians and Muslim immigrants from the Levant, Africa (who migrated in ancient Islamic times). Consequently, much intermarriage has reinforced genetic drift and gene flow to Saudi people, which might influence the development of various carcinomas, particularly urinary BC. The countries within the Arabian Peninsula region have experienced rapid economic growth over the past few decades, resulting in major changes in the population's lifestyle. As in other Arab Gulf countries, the overall rate of consanguinity in Saudi community shows that 57.7% of the families are consanguineous; the most frequent marriages are first-degree cousin marriages (28.4%) followed by distant relative marriages (15.2%) and second-cousin marriages (14.6%) [25]. These higher rates result in increasing heterozygosity for several autosomal recessive genetic diseases [26] and even enhance the heritability of some complex multifactorial disorders [27, 28]. In an Egyptian study, parents' consanguinity (50.8%; *P* < 0.001) was shown to have a more prominent role than bilharziasis and smoking in the development of BC [29].

Despite a significant amount of available genetic information on susceptibility to BC, most reports are from Caucasian and Asian populations [30–34]. Data on genetic linkages and their relevance to the development of BC are still limited in Saudi patients [35].

We hypothesize that genetic variants within the GSTM1/GSTT1, CYP450, TP53, MTHFR, and MTRR genes might be each linked alone or linked in combination with the occurrence of UBC. The present study was carried out to examine the associations of GSTM1, GSTT1, rs4646903 and rs1048943, rs1042522, rs1801133, and rs1801394 genetic loci with the risk of UBC tumors in a Saudi population.

## 2. Subjects and Methods

### 2.1. Ethics Statement

This study was approved by the Institutional Biomedical Ethics Committee at Faculty of Medicine, Umm Al-Qura University (reference #HAPO-02-K-012), licensed from the National Committee of Medical & Bioethics, KACST, Riyadh (http://bioethics.kacst.edu.sa/About.aspx?lang=en-US). Details of the study were told to participants, and written informed consent was obtained before enrollment.

### 2.2. Study Population

The study included 52 patients (aged 49–90 years) diagnosed with UBC who were admitted to the Urology Department at King Abdullah City Hospital in Mecca between June 2014 and January 2016. Epidemiologic and clinical characteristics of gender, age at diagnosis, family history of cancer, cigarette smoking habits, alcohol consumption, pathologic tumor stage, tumor grade, and metastasis were recorded for each patient for statistical analyses. The management of the disease—through intravesical* bacillus Calmette-Guérin* (BCG), immunotherapy, a conservative TURBT-BCG therapy (injection), chemotherapy, chemoradiotherapy, or radical cystectomy—was also recorded. After having done a TURBT, only UBC with high-grade UBC cases was treated with BCG. Patients who previously had cancer, radiotherapy and chemotherapy, or metastasized cancer from non-Saudi or unknown origins were excluded. Patients with immune disease or any histopathologic diagnosis other than UBC were also excluded. Healthy individuals (*n* = 104) having no evidence of any clinical phenotypes of malignancies or immune disease were selected as controls (44–89 years) in a routine follow-up at governmental hospitals in Mecca.

### 2.3. DNA Isolation

DNA samples were extracted from buccal mucosa using the Oragene.DNA-OGR-575 Kit (DNA Genotek Inc., Ottawa, ON, Canada) with some modifications. The full buccal cells were collected within 30 minutes, and the Oragene tube was capped immediately. The cells were incubated with the OGR-lysis buffer in a water bath at 53°C to release the DNA, which was then precipitated by ethanol and dissolved in elution buffer [36].

### 2.4. Duplex PCR of GSTM1 and GSTT1 Loci

Previously reported primers for the* GSTM1 *and* GSTT1* loci were simultaneously amplified in a single assay and detect the 215-bp and 480-bp fragments, respectively [37]. The PCR amplicon was then analyzed by electrophoresis on a 2% ethidium bromide-stained NuSieve agarose gel (BMA Bioproducts, Rockland, ME). Each sample was run in duplicate, and a positive control was used for each variant. The genotypes of all DNA samples were reassessed twice to confirm the results and ensure reproducibility.

### 2.5. TaqMan Genotyping Analysis

We adopted TaqMan real-time PCR assays to genotype the selected five SNPs—rs4646903 (C_8879532_20), rs1048943 (C_25624888_50), rs1042522 (C_2403545_10), rs1801133 (C_1202883_20), and rs1801394 (C_3068176_10)—in cases and controls using a 7500 Fast Dx Real-Time PCR System (Applied Biosystems, Life Technologies Inc., USA). Eight negative controls and 104 DNA samples (cases and controls) were included in a 96-well plate to ensure the accuracy of genotyping results. We also repeatedly genotyped 10% of the samples, and the results were 100% concordant.

### 2.6. Databases of Examined SNPs

The functional consequences of the examined SNP markers were predicted with the web servers; Sorting Intolerant From Tolerant (SIFT; http://sift.jcvi.org/), and Polymorphism Phenotyping (PolyPhen2; http://genetics.bwh.harvard.edu/pph2/).

### 2.7. Statistical Analysis

Deviations from Hardy-Weinberg equilibrium (HWE) were examined in the study group for healthy controls* via* chi-squared testing using online software (http://www.oege.org/software/hwe-mr-calc.shtml).   Student's *t*-test and chi-square test were used to compare demographic and clinical characteristics including age, gender, cigarette smoking, alcohol consumption, tumor grade, pathologic stage, and clinical management. Associations of polymorphisms with UBC were evaluated using ORs (odds ratios), 95% CIs (confidence intervals), and a general* z*-test with MedCalc Statistical software version 16.4.3 (MedCalc Software bvba, Ostend, Belgium; https://www.medcalc.org; 2016). A two-sided *P* value less than 0.05 was considered to indicate statistical significance for all analyses. We used the *G*^*∗*^Power Software (Franz Faul, Universität Kiel, Germany, ver. 3.1.9.2) (http://www.psycho.uni-duesseldorf.de/abteilungen/aap/gpower3/download-and-register/) to perform a priori power analysis to calculate sufficient sample sizes to achieve adequate power for* z*-testing of two independent proportions. A priori sample size estimations were performed using known information on the common allele frequencies in UBC cases and healthy controls, a criterion probability of *α* = 0.05, and a power sensitivity of 80%.

## 3. Results

### 3.1. Characteristics of the Study Population

For the study, 52 eligible Saudi individuals with UBC (48 men : 4 women; 12 : 1) and 104 healthy controls (92 men : 12 women; 7.7 : 1) were enrolled. The mean age of patients was 61.8 ± 10.63 years, with no significant difference when compared with controls (*P* > 0.05). There was no significant difference between cases and controls in terms of the percentage of current cigarette smokers (80.8% versus 76.9%, resp.; *P* = 0.6). Significant differences (*P* < 0.0001) were found in the proportion of cases with a high tumor grade (35 cases, 67%) and the proportion with a low tumor grade (17 cases, 32.7%). The percentages of UBC cases with specific tumor stages were 59.6% for Ta, 17.3% for T1, and 23.1% for T2 (*P* < 0.0001) (Table 1). The management course of UBC cases was significantly predominant with BCG immunotherapy (76.9%; *z* = 23.8, *P* < 0.0001). Conservative management with TURBT-BCG injection was significantly higher than other types of treatment (80.8% versus 9.6% for radical cystectomy, 5.8% for chemoradiotherapy, and 3.8% for chemotherapy).

### 3.2. Hardy-Weinberg Equilibria of SNPs

All controls were in HWE at the rs4646903 T>C (*χ*^2^ = 3.1, *P* = 0.08), rs1048943 A>G (*χ*^2^ = 0.39, *P* = 0.5), rs1042522 Pro>Arg (*χ*^2^ = 3.0, *P* = 0.08), and MTHFR rs1801133 C>T (*χ*^2^ = 0.72, *P* = 0.4), and MTRR rs1801394 A>G loci (*χ*^2^ = 1.8, *P* = 0.18). HWE could not be tested for the GSTM1 and GSTT1 null genotypes because of the inability of the conventional restriction fragment length polymorphism protocol to separate heterozygous carriers of the deletion polymorphisms.

### 3.3. Allele Frequencies and Genotype Distributions

The frequencies of the GSTM1 null and GSTT1 null alleles were higher in UBC cases than in controls (0.46 versus 0.38; OR = 1.4, 95% CI, 0.9–2.2, *z* = 1.3, and *P* = 0.19 for GSTM1; 0.12 versus 0.08; OR = 1.6, 95% CI, 0.7–3.4, *z* = 1.1, and *P* = 0.27 for GSTT1) (Table 2). Genotypic distribution of the GSTM1 and GSTT1 polymorphisms showed a higher frequency of the null-variants in UBC cases than in controls (46.2% versus 38.5% for the homozygous GSTM1 null genotype and 11.5% versus 7.7% for the homozygous GSTT1 null genotype). There were no differences in the homozygous null genotypes of the GSTM1 and GSTT1 alleles between the UBC cases and the controls (*χ*^2^ = 0.8, *P* = 0.4; *χ*^2^ = 0.6, *P* = 0.4).

The differences between the variant alleles with the cases and controls were not statistically significant for three SNPs: rs4646903 (OR = 0.8, 95% CI, 0.5–1.5, *z* = 0.6, and *P* = 0.6), rs1048943 (OR = 1.4, 95% CI, 0.5–3.4, *z* = 0.7, and *P* = 0.5), and rs1042522 (OR = 0.7, 95% CI, 0.5–1.2, *z* = 1.3, and *P* = 0.2). But there were significant differences in this issue for two SNPs: rs1801133 (OR = 3.6, 95% CI, 1.8–7.1, *z* = 3.7, and *P* = 0.0002) and rs1801394 (OR = 4.1, 95% CI, 2.5–6.7, *z* = 5.5, and *P* < 0.0001) (Table 2).

There were strong, statistically significant differences between the genotypes of cases and controls for the MTHFR rs1801133 SNP (*χ*^2^ = 14.4, *P* = 0.0007) and the MTRR rs1801394 SNP (*χ*^2^ = 35.6, *P* = 0.00001). For the rs1801394 SNP, there was an absence of AA wild-type carriers among UBC cases but a 38.5% prevalence of AA wild-type carriers among controls (*z* = 26.8, *P* = 0.0001). GG-variants were more frequent among UBC cases than among controls (38.5% versus 9.6%; *z* = 18.5, *P* = 0.0001). For the rs1801133 SNP, there were significant differences between cases and controls for the CC wild-type (*z* = 8.8, *P* = 0.003) and TT-variants (*z* = 9.6, *P* = 0.001) genotypes. On the other hand, there were no linkage disequilibria between the rs4646903 and rs1048943 SNPs and UBC (*P* = 0.4 and *P* = 0.1, resp.) (Table 2). For the rs1042522 SNP, the homozygote Pro/Pro conferred a double risk with a significant difference in cases compared with controls (30.8% versus 15.4%; *P* = 0.03), but the homozygote Arg/Arg had no effect on risk (*P* = 1.0). None of the genotypes of the rs4646903 SNP or rs1048943 SNP were linked to the risk of UBC (*P* = 0.4 and *P* = 0.1, resp.).

### 3.4. Genotypic Combinations

GSTM1/GSTT1 genotypic combinations were significantly different in cases and controls (*χ*^2^ = 10.3, *P* = 0.006), with the “−,−/+,+” combination being the most prevalent in UBC cases (53.8% versus 38.5%; *χ*^2^ = 6.6, *P* = 0.01). There were no cases or controls with the combined null genotype “−,−/−,−". In contrast, the “+,+/+,+” combination was more prevalent in controls than in cases (53.8% versus 34.6%), with a strong statistical significance (*χ*^2^ = 10.2, *P* = 0.001). These outcomes might suppose the pathological role of the GSTM1 null genotype in the risk of the disease. There was a significant difference in the genotypic combinations of rs4646903 T>C/rs1048943 A>G in cases versus controls (*χ*^2^ = 13.9, *P* = 0.003), including a significant difference in the homozygous variant (CC/GG), which was absent in controls but seen in 3.8% of cases (*χ*^2^ = 4.0, *P* = 0.047). There was also a strong significant difference regarding rs1801133 C>T/rs1801394 A>G combinations in cases versus controls (*χ*^2^ = 20.4, *P* = 0.0004), with the absence of CT/GG in controls (*χ*^2^ = 8.3, *P* = 0.004) (Table 3).

### 3.5. Effect of Smoking on Allelic Distributions

Table 4 shows the differential allelic frequency of the examined SNPs in current smokers with UBC (*n* = 42, 80.8%). The variant alleles of GSTM1 null, rs1042522 Arg, and MTRR rs1801394G were more prevalent in current smokers with UBC disease (52.4%, 52.5%, and 64.3%, resp.) than were the wild-type alleles. However, the difference was only statistically significant for the rs1801394G allele (*χ*^2^ = 13.7, *P* = 0.0002). Significant differences were shown for other examined SNPs but linked dominantly with the wild-type alleles (Table 4).

### 3.6. Prediction of Examined SNP Markers

The SIFT and PolyPhen2 were utilized to predict amino acid substitution effects and to predict damaging effects. The SIFT algorithm predicted the four nonsynonymous SNPs; rs1048943 (p.I462V), rs1042522 (p.P72R), rs1801133 (p.A222V), and rs1801394 (p.I22M) were deleterious to effect protein function with scores, 0.16, 0.16, 0.03, and 0.01, respectively. The PolyPhen2 also predicted that all these SNPs were possibly damaging with a scoring range 0.265 to 0.962, except for the CYP1A1 rs1048943 polymorphism which showed benign phenotypes (Table 5).

## 4. Discussion

This hospital-based case-control study presents the first investigation, to our knowledge, of potential associations between the GSTM1 null, GSTT1 null, rs4646903 T>C, rs1048943 A>G, rs1042522 Pro>Arg, rs1801133 C>T, and rs1801394 A>G polymorphisms and UBC susceptibility in a Saudi population.

We found no deviation in our controls from HWE for all examined SNPs. The observed frequency of the MTRR rs1801394 G/G homozygote was higher than expected (38.5% versus 9.6%). The duplex PCR could not differentiate between the “+,+” homozygote or the “+,−" heterozygote carrying the null allele. However, we might consider the individual with a heterozygous genotype “+,−" as a homozygote with “+,+” genotype. This could result in a weaker impact of the null alleles on UBC risk.

Overall, our results provide strong evidence of associations between two SNPs—rs1801133 C>T and rs1801394 A>G—and risk of UBC in this population. The variants rs1801133T and rs1801394G can potentially affect the risk of UBC (*P* = 0.009 and *P* < 0.0001, resp.). None of the GSTM1 null, GSTT1 null, rs4646903, rs1048943, or TP53 rs1042522 variant was linked to risk of the disease in this study. Our findings regarding combined GSTM1/GSTT1 genotypes support other studies showing that the GSTM1 null genotype is significantly associated with increased UBC risk, irrespective of the GSTT1 genotype status (−,−/+,+; *P* = 0.01). The inherited GSTM1/GSTT1 null allele combination (−,−/−,−) was absent in our cases, which is inconsistent with studies in Turkish populations [38]. Sometimes, it is difficult to expect how the GSTM1 and GSTT1 enzymes may potentially influence BC susceptibility, as they have multifunctional roles in metabolic pathways.

Eight case-control studies have produced conflicting results regarding the association between CYP1A1 polymorphisms and BC susceptibility [7, 38–43]. However, most of these studies have observed no significant association with BC risk [44]. Among them, a few evaluated the association between the rs4646903 locus and BC risk and found slight trends toward association in German, Chinese, and North Indian populations [39, 40, 43, 44]. Despite the weak impact of the separate CYP SNPs (rs4646903 and rs1048943) in the present study, the combined genotype has a significant linkage with UBC (*P* = 0.003).

The present study found that the Pro/Arg heterozygotes of TP53 rs1042522 were prevalent among both cases and controls, with no significant difference between the two (42.3% versus 57.7%, resp.; *P* = 0.07). However, some studies support a passive risk of BC among Arg/Arg carriers in the Saudi population [45]. Also, in our study, homozygosity of Pro72 had a double effect on risk for UBC cases when compared with controls (*P* = 0.03), but homozygosity 72Arg had no effect on our Saudi UBC risk (*P* = 1.0). It has been found that Pro72 homozygotes can improve survival after development of cancer compared with 72Arg homozygotes [46]. It is known that 72Arg increases the ability of TP53 to translocate to mitochondria and induce cell death, as Pro72 exhibits lower apoptotic potential but increases cellular arrest in G1 of the cell cycle [46]. Previous studies have demonstrated that the functional polymorphisms of the heterozygous Pro/Arg are associated with susceptibility to a variety of cancers, such as thyroid cancer, lung cancer, and cervical cancer [47–49]. Other studies have reported that Pro/Pro and Arg/Arg homozygosity may increase susceptibility to thyroid cancer and cervical cancer [45, 49, 50].

The variant homozygotes of the rs1801133 and rs1801394 SNPs can clearly influence the risk of UBC (*P* = 0.001 and *P* = 0.0001, resp.). For rs1801133TT, our data outcomes are consistent with those in German (Caucasian), Chinese, Netherlander, and Turkish populations [15, 51–56]. In contrast, a meta-analysis found no association between the 677C>T polymorphism and BC in the Chinese population [57]. Besides, several studies reported no linkage disequilibrium in 677C>T with BC risk in populations from the United States, Spain, Tunis, Iran, and Taiwan [16, 18, 58–62]. Few studies have dealt with the rs1801394 SNP and those that have inconsistent results regarding association with BC [16, 18, 19].

Despite a limited trend of associations between some SNPs examined in this Saudi sample group and risk of UBC, this work clearly showed that genotypic combinations of GST (GSTM1/GSTT1) and CYP (rs4646903/rs1048943) could reinforce statistical linkage disequilibrium with the disease (*P* = 0.006 and *P* = 0.003, resp.). Moreover, genes involved in folate metabolism (rs1801133/rs1801394) clearly exhibit strong linkage with UBC (*P* = 0.0004), and this outcome is consistent with previous studies regarding the same combined genotypes of MTHFR/MTRR loci in a Tunisian population [16].

The incidence of BC in men is higher in the northwestern part of West Asia (Turkey and Lebanon) than in other parts, and cigarette smoking is proposed as the most important risk factor for BC in these countries [63, 64]. Among the current smokers with UBC in our study, the variant alleles of GSTM1 null, TP53 rs1042522Arg, and MTRR rs1801394G were more frequent than the wild-type alleles. Slightly more smokers with UBC had GSTM1 null alleles than wild-type alleles. Bell et al. [65] have previously stated that the association among the GSTM1 null-variant, cigarette smoking, and BC has been controversial, indicating higher risks in smokers due to lack of GSTM1. Meta-analyses and pooled studies have found no or only weak evidence for an association between the GSTM1 null-variant and smoking habits [66–69]. Rothman et al. [69] have reported an even higher OR for nonsmokers than for ever-smokers (1.71 versus 1.47). However, only the variant G allele of the rs1801394 SNP was strongly associated with the disease (*P* = 0.0002). Clearly, there was no effect of smoking habits associated with the GSTT1 null, rs4646903 T>C, rs1048943 A>G, and MTHFR 677C>T SNPs.

Occupational exposure (e.g., to diesel) has also been suggested as a risk factor for BC [64]. However, any effect of occupational history in our study was clearly passive, as these UBC patients were working in business affairs (92.3%) or as house wives (7.7%). In one study, BC patients exposed to polycyclic aromatic hydrocarbons (i.e., people with occupational histories in coal, iron, and steel industries) presented with high percentages of GSTM1 null genotypes [70, 71]. Fortunately, another study suggested that polycyclic aromatic hydrocarbons had no effect on GSTM1 once these industries had been closed, and the GSTM1 genotypes were equal in both cases and controls (GSTM1 “−” = 52%) [72].

Based on the functional prediction of SIFT and PolyPhen2 web links, three examined SNPs (TP53 rs1042522, MTHFR rs1801133, and MTRR rs1801394) are suggested to have serious effects of damaging on the functions and risks to UBC polymorphic phenotypes. But the CYP rs1048943 SNP shares a benign prediction in this issue.

Conflicting results in pinning down a genetic association for urinary BC are not uncommon, as poor replication of studies can arise from several different factors. Following are a few example of how this can happen.* First*, when compared with our study, some studies have had populations with admixed ethnicities, different sources of BC/control subjects, or small sample sizes, which would lessen the strength of the overall results to be consistent with our Saudi population. In addition, the literature includes other pathological types of cancer (e.g., squamous or adenocarcinoma) in addition to UBC, which would undoubtedly influence the outcomes of association of the genetic markers to BC. However, the present study did focus only on the UBC rather than other types which consequently strengthen the reliability of our outcomes of associations with the disease.* Second*, various technologies have been used in previous studies to examine and discriminate different genotypes within a specific SNP, resulting in a broad range of false-negative or false-positive genotypic distributions. Our study primarily used a conventional PCR protocol, as in other publications, to identify the GSTM1 and GSTT1 null-variants; hence, the slight trends of association of the null alleles with UBC risk were weaker than expected in our outcomes.* Third*, our post hoc statistical analyses for the MTHFR rs1801133 and MTRR rs1801394 SNPs revealed powers of 39.8% and 98%, resp., among our 156 participants. Recruiting 372 and 70 participants for these SNPs, respectively, would be sufficient to reach a power of 80%. Usually smaller sample sizes lead to lower post hoc power of detection. According to our “a priori” calculations, we would be in need of sampling sizes of 596, 882, 2842, 2554, and 611 in both cases and controls for GSTM1, GSTT1, rs4646903, rs1048943, and rs1042522, respectively, to get 80% power of detection. Overcoming limitations such as these can be difficult, but meta-analysis may offer a feasible option, as it permits surveying a wide set of subjects, thereby enhancing post hoc power and allowing for a more broadly based analysis of previously available data. Recruiting more participants within a reasonable time frame from a single hospital or clinical center would have been challenging in our study, but replication of our results through larger, multicenter genetic association studies will be of interest.

Recently, Li and Chen [43] utilized the available omics data of next-generation sequencing to evaluate the miRNA regulatory network biomarkers (e.g., miR-200a, miR-200b) to investigate BC mechanisms and design multiple drug combinations for treating BC. Moreover, the role of miRNAs in several BC scenarios has been described by Matullo et al. [73], suggesting they could become relevant clinical markers. Ward et al. [34] have utilized next-generation sequencing to amplify DNA from the urine of BC patients and have detected mutations with 70% sensitivity; 54% of the mutations were in TERT, 28% in FGFR3, 13% in PIK3CA, 13% in TP53, 5% in RXRA, and 1% in HRAS mutations. Some additional genomic alterations have included TP53 (54%) and RB1 (17%) [74].

## 5. Conclusions

In conclusion, this study presents evidence that the MTHFR rs1801133 and MTRR rs1801394 variants influence genes involved in folate metabolism, remarkably increasing the risk of UBC in the Saudi population. Moreover, the GSTM1, GSTT1, CYP (rs4646903 and rs1048943), and TP53 rs1042522 Pro>Arg variants showed a slight trend of linkage disequilibria but can interact additively to increase the risk of UBC. So far, these outcomes should be taken with caution, as these SNPs do not act alone to explain such complex multifactorial malignancies. More linkage outcomes based on a whole-genome approach instead of a single gene or a few candidate genes may be useful for discovering new genes associated with susceptibility to BC. The association between the GSTM1 variant and current smokers with UBC has been controversial, indicating higher risks in smokers due to the GSTM1 null-variant; however, no potential role was found for cytochrome P450, MTHFR 677C>T, or MTRR 66A>G in smoking habits in this study. These associations or linkages are predictive and intriguing, but they need to be confirmed by additional studies. Ongoing analyses of UBC patient DNA using next-generation sequencing protocols are being performed.

## Figures and Tables

**Table 1:** Epidemiologic and clinical characteristics in UBC cases.

Parameter	UBC cases *n* = 52	*z* (95% CI), *P* value
Age at examination (range, years)	46–90	
Mean age ± SD (years)	61.8 ± 10.63	38.5 (58.8–64.8)^a^
Gender, males (%)^b^	48 (92.3)	28.9 (81.5–97.9)^c^
Cigarette smoking (current)^b^	42 (80.8)	25.1 (67.5–90.4)^c^
Alcohol consumption (yes)	—	—
*Tumor grade*:		
Low-grade^b^	17 (32.7)	9.2 (20.3–47.1)^c^
High-grade^b^	35 (67.3)	20.6 (52.9–79.7)^c^
*Pathologic stage*:		
pTa^b^	31 (59.6)	18.1 (45.1–73.0)^c^
pT1^b^	9 (17.3)	4.1 (8.2–30.3)^c^
pT2^b^	12 (23.1)	6.0 (12.6–36.9)^c^
*Metastasis* (yes):^b^	0 (0.0)	
*Management*:		
BCG^b^ (yes)^a,d^	40 (76.9)	23.8 (63.1–87.5)^c^
Conservative therapy^b,e^	42 (80.8)	25.1 (67.5–90.4)^c^
Radical cystectomy^b^	5 (9.6)	1.5 (3.2–21.0) (*P* = 0.1)^f^
Chemoradiotherapy^b^	3 (5.8)	0.3 (1.2–16.0) (*P* = 0.8)^f^
Chemotherapy^b^	2 (3.8)	0.4 (0.5–13.1) (*P* = 0.7)^f^

^a^ Student's *t*-test. Values are mean ± standard deviation (SD).

^b^Number of patients, with percentages in parentheses.

^c^Very highly significant difference (*P* < 0.0001).

^d^BCG, bacillus Calmette-Guérin, a weakened bacterium intravesically introduced via a catheter.

^e^“Conservative therapy,” TURBT plus BCG.

^f^No significant difference (*P* > 0.05).

**Table 2:** Genotype distributions and allele frequencies of examined SNPs in UBC cases.

SNP ID	Cases	Controls	*χ* ^2^ (*P* value)^a^	*χ* ^2^ (*P* value)^b^ OR (95% CI); *z* (*P* value)^c^
	*n* (%)	*n* (%)		
*GSTM1*:				
“+/+”	28 (53.8)	64 (61.5)	0.8 (0.4)^a^	0.8 (0.4)^b^
“−/−”	24 (46.2)	40 (38.5)	0.8 (0.4)^a^	
“+”	56 (0.54)	128 (0.62)		1.4 (0.9–2.2); 1.3 (0.19)^c^
“−”	48 (0.46)	80 (0.38)		
*GSTT1*:				
“+/+”	46 (88.5)	96 (92.3)	0.6 (0.4)^a^	0.6 (0.4)^b^
“−/−”	6 (11.5)	8 (7.7)	0.6 (0.4)^a^	
“+”	92 (0.88)	192 (0.92)		1.6 (0.7–3.4); 1.1 (0.27)^c^
“−”	12 (0.12)	16 (0.08)		
				
*CYP1A1 T6235C (rs4646903)*:				
TT	34 (65.4)	60 (57.7)	0.8 (0.4)^a^	1.7 (0.4)^b^
TC	16 (30.8)	42 (40.4)	1.4 (0.2)^a^	
CC	2 (3.8)	2 (1.9)	0.5 (0.5)^a^	
T	84 (0.81)	162 (0.78)		0.8 (0.5–1.5); 0.6 (0.56)^c^
C	20 (0.19)	46 (0.22)		
*CYP1A1 A4889G (rs1048943)*:				
AA	46 (88.5)	92 (88.5)	0.0 (1.0)^a^	4.5 (0.1)^b^
AG	4 (30.8)	12 (11.5)	8.7 (0.003)^a^	
GG	2 (26.9)	0 (0.0)	30.5 (<0.0001)^a^	
A	96 (0.92)	196 (0.94)		1.4 (0.5–3.4); 0.7 (0.5)^c^
G	8 (0.08)	12 (0.06)		
*TP53 Pro72Arg (rs1042522)*:				
Pro/Pro	16 (30.8)	16 (15.4)	5.0 (0.03)^a^	5.6 (0.06)^b^
Pro/Arg	22 (42.3)	60 (57.7)	3.3 (0.07)^a^	
Arg/Arg	14 (26.9)	28 (26.9)	0.0 (1.0)^a^	
Pro	54 (0.52)	92 (0.44)		0.7 (0.5–1.2); 1.3 (0.2)^c^
Arg	50 (0.48)	116 (0.56)		
*MTHFR C677T (rs1801133)*:				
CC	33 (63.5)	88 (84.6)	8.8 (0.003)^a^	14.4 (0.0007)^b^
CT	14 (26.9)	16 (15.4)	2.9 (0.09)^a^	
TT	5 (9.6)	0 (0.0)	10.2 (0.001)^a^	
C	80 (0.77)	192 (0.88)		3.6 (1.8–7.1); 3.7 (0.0002)^c^
T	24 (0.23)	16 (0.12)		
*MTRR A66G (rs1801394)*:				
AA	0 (0.0)	40 (38.5)	26.8 (<0.0001)^a^	35.6 (0.00001)^b^
AG	32 (61.5)	54 (51.9)	1.3 (0.3)^a^	
GG	20 (38.5)	10 (9.6)	18.5 (<0.0001)^a^	
A	32 (0.31)	134 (0.64)		4.1 (2.5–6.7); 5.5 (<0.0001)^c^
G	72 (0.69)	74 (0.36)		

“+”: present allele; “−”: null allele; “−,−”: homozygous null genotype; “+,+”: homozygous present genotype; OR: odds ratio; CI: confidence interval.

^a^Statistical difference between two similar genotypes in cases and controls.

^b^Statistical difference between genotypes of a specific SNP in cases compared with controls.

^c^Comparison of allele frequencies of a specific SNP.

**Table 3:** Genotypic combinations of examined SNPs showing risk of UBC in cases and controls.

Genotypic combination	Cases *n* (%)	Controls *n* (%)	*χ* ^2^ (*P* value)^a^	*χ* ^2^ (*P* value)^b^
*GSTM1/GSTT1*:				
+,+/+,+	36 (34.6)	112 (53.8)	10.2 (0.001)	
−,−/+,+	56 (53.8)	80 (38.5)	6.6 (0.01)	10.3 (0.006)
+,+/−,−	12 (11.5)	16 (7.7)	1.2 (0.3)	
−,−/−,−	0 (0.0)	0 (0.0)	NA	NA
*rs4646903 T>C/ rs1048943 A>G*:				
TT/AA	72 (69.2)	120 (57.7)	3.9 (0.04)	
TC/AG	8 (7.7)	24 (11.5)	1.1 (0.3)	
TC/AA	20 (19.2)	64 (30.8)	4.7 (0.03)	13.9 (0.003)
CC/GG	4 (3.8)	0 (0.0)	4.0 (0.047)	
*rs1801133 C>T/ rs1801394 A>G*:				
CC/AA	0 (0.0)	8 (3.8)	4.2 (0.04)	
CC/AG	56 (53.8)	120 (57.7)	0.4 (0.5)	
CC/GG	20 (19.2)	32 (15.4)	0.7 (0.4)	
CT/AA	0 (0.0)	8 (3.8)	4.2 (0.04)	20.4 (0.0004)
CT/AG	20 (19.2)	40 (19.2)	0.0 (1.0)	
CT/GG	8 (7.7)	0 (0.0)	8.3 (0.0001)	

“+”: present allele; “−”: null allele; “+,+”: homozygous present genotype; “−,−”: homozygote null genotype; OR: odds ratio; CI: confidence interval.

^a^Statistically significant difference between a specific combined genotype in cases and controls.

^b^Statistically significant difference between combined genotypes in cases compared with controls.

**Table 4:** Effect of SNP allele percentages on phenotypes of 42 smokers with UBC.

SNP ID	Wild-type allele *n* (%)	Variant allele *n* (%)	*χ* ^2^ (*P* value)
GSTM1 (+,−)	40 (47.6)	44 (52.4)	0.4 (0.5)
GSTT1 (+,−)	72 (85.7)	12 (14.3)	85.1 (<0.0001)
rs4646903 T>C	70 (83.3)	14 (16.7)	74.1 (<0.0001)
rs1048943 A>G	78 (92.9)	6 (7.1)	122.9 (<0.0001)
rs1042522 Pro>Arg	40 (47.5)	44 (52.5)	0.4 (0.5)
rs1801133 C>T	74 (88.1)	10 (11.9)	97.0 (<0.0001)
rs1801394 A>G	30 (35.7)	54 (64.3)	13.7 (0.0002)

**Table 5:** SIFT and PolyPhen2 functional predictions of SNP markers.

SNP ID	Allele	Amino acid position	SIFT			PolyPhen2	
			Score	Prediction	Confidence	Score	Prediction
rs1048943	T>C	p.I462V	0.16	Tolerated	High	0.18	Benign
rs4646903	A>G	NA^*∗*^	—	—	—	—	—
rs1042522	G>C	p.P72A	0.16	Tolerated	High	0.265	Possibly damaging
rs1801133	C>T	p.A222V	0.03	Damaging	High	0.235	Possibly damaging
rs1801394	A>G	p.I22M	0.01	Damaging	High	0.962	Possibly damaging

SIFT, Sorting Intolerant From Tolerant (http://sift.jcvi.org/); PolyPhen2, Polymorphism phenotyping (http://genetics.bwh.harvard.edu/pph2/).

^*∗*^NA: not applicable as it is in the noncoding 3′ flanking untranslated region. Low confidence means that the protein alignment does not have enough sequence diversity. Because the position artificially appears to be conserved, an amino acid may incorrectly be predicted to be damaging.
